# The validity and reliability of the Turkish version of the Physical Activity Scale for Individuals with Physical Disabilities (PASIPD)

**DOI:** 10.3906/sag-1901-113

**Published:** 2019-12-16

**Authors:** Kardem ULAŞ, Semra TOPUZ, Gönül DİNÇ HORASAN

**Affiliations:** 1 Department of Physiotherapy, Vocational School of Health Services, İzmir University of Economics, İzmir Turkey; 2 Department of Physiotherapy and Rehabilitation, Faculty of Physiotherapy and Rehabilitation, Hacettepe University, Ankara Turkey; 3 Faculty of Medicine, İzmir University of Economics, İzmir Turkey

**Keywords:** Disabled, physical activity, level, impairment

## Abstract

**Background/aim:**

The aim of this study is to assess the validity and reliability of Turkish translation of the Physical Activity Scale for Individuals with Physical Disabilities (PASIPD) in a disabled Turkish population.

**Materials and methods:**

Following the translation protocol of the PASIPD, the Turkish version of the PASIPD, Short Form-36, and International Physical Activity Questionnaire were administered to 198 developmentally and physically disabled individuals.

**Results:**

The Turkish version of the PASIPD was found to be reliable. The domains of the Turkish version of the PASIPD were also found to be valid. Four factors were obtained from the questionnaire. The ICC was 1.0 since all the respondents reported the same answers in the test and retest. The Cronbach α for the PASIPD was 0.60.

**Conclusion:**

The Turkish version of the PASIPD survey is valid and reliable for developmentally and physically disabled Turkish individuals and professionals can use it to assess physical activity level.

## 1. Introduction

Physical activity is defined as activities that increase both cardiorespiratory functions and energy consumption during daily life [1]. Almost all physically disabled individuals consume more energy than healthy individuals during personal and social physical activities in their daily lives [2–4]. An increased disability level has been shown to cause a decrease in physical activity levels [5–9].

Accelerometers, pedometers, heart rate monitors, and various types of questionnaires can measure physical activity levels [10,11]. In the literature, the most commonly used method is the questionnaire format, for reasons of accessibility and simplicity [12]. Most of these have targeted healthy individuals, which makes those questionnaires inadequate to measure the levels of physical activity of disabled individuals [13]. For the optimal well-being of the disabled population, it is crucial to measure the physical activity level in every aspect [14]. Therefore, Washburn et al. developed a questionnaire to measure the physical activity level in every dimension, including mild to severe physical activities, gardening, heavy household chores, and caring for another person among physically disabled individuals [14,15]. 

Taking into consideration the fact that there are a limited number of questionnaires in the Turkish language that measure the physical activity levels of disabled individuals, the present study aimed to determine a valid questionnaire for clinicians to measure the activity levels of physically disabled individuals in a Turkish population.

## 2. Materials and methods

### 2.1. Participants

The study included 198 physically disabled patients (>18 years old) from two metropolitan cities in Turkey, Ankara and İzmir. The subjects completed the questionnaires by one of three communication methods: through telephone, e-mail, or face-to-face interviews in the Department of Physiotherapy at the Faculty of Health Sciences of Hacettepe University in Ankara. All the participants read and signed a written informed consent form. The study was approved by the Hacettepe University Non-interventional Clinical Research Ethics Board (GO 17/378). 

### 2.2. Questionnaires

#### 2.2.1. Physical Activity Scale for Individuals with Physical Disabilities (PASIPD)

The PASIPD is a modification of the Physical Activity Scale for the Elderly (PASE), which was adapted to be suitable for physical disabilities by Washburn et al. [16]. There are 5 identified factors in this scale: Factor 1, home repair, lawn and garden work; Factor 2, housework; Factor 3, vigorous sports and recreation; Factor 4, light/moderate sports and recreation; and Factor 5, occupation. The PASIPD comprises 13 items: 6 on leisure time, including walking and wheeling outside the home, and exercising with light to moderate and strenuous sport for recreation; 6 on household tasks, including light and heavy housework, outdoor gardening, repairs, and caring for another person; and 1 on occupational activity. For all items, the subject is asked to recall the number of days in the past 7 days when these activities have been undertaken, from never, seldom (1–2 days/week), sometimes (3–4 days/week), or often (5–7 days/week), and on average how many hours per day (<1 h, ≥1 but <2 h, 2–4 h, >4 h). For the occupational item, the hours are per day (<1 h, ≥1 but <4 h, ≥5 but <8 h, ≥8 h). The score for the PASIPD is calculated by multiplying a MET value, indicated by the developers, with the average hours per day for each item related to the intensity of the activity, with a total of 2 to 13 [16].

#### 2.2.2. Short Form-36 (SF-36)

The Turkish version of the SF-36 was used [17]. The SF-36 was developed by Ware et al. and evaluates: physical functioning (PF), role limitations due to physical problems (RP), bodily pain (BP), general health perception (GH), vitality (VT), social functioning (SF), role limitations due to emotional problems (RE), and mental health (MH) [17]. 

#### 2.2.3. International Physical Activity Questionnaire (IPAQ)

The Turkish version of the IPAQ consists of vigorous physical activity (football, basketball, aerobic, vigorous cycling, heavy lifting, etc.) duration (min), moderate physical activity (carrying light loads, moderate cycling, folklore, dance, bowling, table tennis, etc.) duration (min), walking, and total daily sitting duration. Vigorous and moderate physical activity, walking durations in accordance with basal metabolic rate MET, and total physical activity score (MET min/week) can be calculated [18]. 

### 2.3. Translation into Turkish and cultural adaptation of the PASIPD

After receiving permission from the developers of the PASIPD, the translation process began with forward translations into Turkish, grammar corrections and adaptation, back-translations, revisions, and consensus by the researchers. 

The PASIPD was translated into Turkish by two independent native Turkish speakers, one of whom was aware of the purpose while the other was unaware. For inconsistencies, both Turkish translations were compared and checked for the adaptations that were made. The questionnaire was then blindly and independently retranslated into English by two native English speakers. The English translations were compared with the original and checked for inconsistencies. The Turkish version was then reviewed by a bilingual researcher [19,20].

During the translation and adaptation process, some changes were made. In questions number 3 and 4, the examples of bowling, doubles tennis, golf with or without using a cart, and ballroom dancing were removed as these kinds of activities are rare in Turkey. However, the rest of the examples were found to be adequate for the descriptions of the physical activity levels.

### 2.4. Test and retest reliability

To quantify the reliability of the Turkish version of the PASIPD, each participant was asked to complete it twice (1–3 days apart).

### 2.5. Validity

To quantify the validity of the Turkish version of the PASIPD, the Short Form-36 (SF-36) and International Physical Activity Questionnaire (IPAQ) were used. 

### 2.6. Data analysis

All statistical analyses were conducted using SPSS 21 for Windows. The statistical signiﬁcance of differences in both PASIPD total and subcategory scores between the groups were analyzed with analysis of variance (ANOVA) and Student’s t-test for independent samples. Post hoc comparisons were used to define group differences. The test and retest reliability was analyzed using intraclass correlations (ICCs) and Spearman’s correlation coefficients [21]. 

#### 2.6.1. Psychometric analyses

##### 2.6.1.1. Validity

Differentiation between the groups and factor analyses were the primary approaches used in the study. Validity coefficients were r ≥ 0.81–1.0, excellent; 0.61–0.80, very good; 0.41–0.60, good; 0.21–0.40, fair; and 0–0.20, poor [21].

##### 2.6.1.2. Reliability

Cronbach’s alpha has been used as an internal consistency coefficient. A Cronbach alpha coefficient higher than 0.7 (r ≥ 0.7) has been accepted as very good. Internal consistency analyses were repeated by calculating “if item deleted”. Alpha values were calculated for each of the items separately that showed the individual contributions of the items to the overall internal consistency of the scale. These analyses indicated that the items of the scale contributed to the overall reliability [21].

##### 2.6.1.3. Exploratory factor analysis (EFA)

An ERA was performed in this study. The analysis was performed using principal axis factoring with varimax rotation on the correlations of the observed variables. The Kaiser–Mayer–Olkin test (KMO ≥ 0.5) and Bartlett criterion (P < 0.05) were used to test the suitability of the variables in the factor analysis as well as to test the sample size [22].

## 3. Results

### 3.1. Sample description

From the 198 participants, the following disabilities were reported: amputee (n: 106), hemiplegia (n: 24), CP (n: 4), auditory impairment (n: 64). The descriptive characteristics of the 106 male and 92 female participants are presented in Table 1. 

**Table 1:** Descriptive characteristics by sex.

	Male (n: 106)	Female (n: 92)	Total (n: 198)
Age (years)	47.3 ± 13.0	46.8 ± 13.3	47.1 ± 13.1
<50 (n: 112)	57.3%	56.0%	56.6%
≥50 (n: 86)	42.7%	44.0%	43.4%
BMI (kg/m^2^)	23.1 ± 3.2	22.9 ± 3.2	23.0 ± 3.2
Weight			
Underweight (BMI <18.5) (n: 8)	4.9%	3.4%	4.0%
Normal (BMI 18.5–24.9) (n: 130)	67.1%	64.7%	65.7%
Overweight (BMI 25–29.9) (n: 60)	28.0%	31.9%	30.3%
Disability			
Amputee (n: 106)	52.4%	54.3%	53.5%
Hemiplegia (n: 24)	13.4%	11.2%	12.1%
CP (n: 4)	1.2%	2.6%	2.0%
Auditory impairment (n: 64)	32.9%	31.9%	32.3%
Total (n: 198)	100.0%	100.0%	100.0%

### 3.2. Reliability

The obtained ICC was 1.0 (P < 0.001). Spearman’s rank correlation coefficients for each question were also 1.0 (P < 0.001).

### 3.3. Factor analysis

Table 2 demonstrates the mean scores for each of the 12 items in the Turkish version of the PASIPD, the correlations between each item and the total score, factor loadings, eigenvalues, percentage of variance, and Cronbach α coefﬁcients. A total of 4 factors were defined as Factor 1, heavy household activities and care for another person (8, 10, 12); Factor 2, sports and occupation (4, 5, 6, 13); Factor 3, gardening (9, 11); and Factor 4, light activities (2, 3, 7). The factors were as follows: heavy household activities and care for another person (25.06%), sports and occupation (11.90%), gardening (10.91%), and light activities (9.77%). The Cronbach α for each of the 4 factors ranged from 0.24 to 0.66 and the total Cronbach α for the PASIPD was 0.60. 

**Table 2:** Item correlation with total score factor loading, eigenvalues, percentage of variance, and Cronbach α for PASIPD.

					Factor loading			
Item	Item no.	Mean ± SD (MET h/day)	Corrected item-total correlation	Item-related Cronbach α	Factor 1: Heavy household chores & care for another person	Factor 2: Sports & occupation	Factor 3: Garden	Factor 4: Light activities
Walk and wheel outside home (not for exercise)	12	0.76 ± 1.92	0.398	0.56	0.795	-	-	-
Strenuous sports and recreation	8	0.83 ± 1.83	0.401	0.56	0.782	-	-	-
Light sports and recreation	10	0.79 ± 2.10	0.302	0.57	0.580	-	-	-
Outdoor garden work	4	0.49 ± 1.33	0.270	0.58	-	0.749	-	-
Light house work	5	1.95 ± 3.68	0.566	0.49	-	0.734	-	-
Work for pay/volunteer	13	7.24 ± 8.27	0.364	0.68	-	0.643	-	-
Heavy house work	6	1.37 ± 2.91	0.606	0.50	-	0.473	-	-
Moderate sports and recreation	11	0.70 ± 2.13	0.172	0.59	-	-	0.795	-
Exercise to increase muscular strength	9	0.64 ± 1.74	0.267	0.58	-	-	0.711	-
Home repair	2	0.75 ± 0.71	0.145	0.60	-	-	-	0.700
Care for another person	7	0.65 ± 0.90	0.195	0.59	-	-	-	0.667
Lawn work and yard care	3	0.42 ± 0.60	– 0.095	0.61	-	-	-	0.427
	PASIPD	16.47 ± 15.80						
	Eigenvalue	-	-	-	3.00	1.42	1.31	1.17
	% variance				25.06	11.90	10.91	9.77
	Cumulative % variance				25.06	36.97	47.89	57.66
	Cronbach α	-	-	-	0.66	0.52	0.46	0.24

Cronbach α for PASIPD: 0.60, KMO: 0.681, Barlett’s test of sphericity: P < 0.001.

### 3.4. Group differentiation

The scores of the group comparisons are shown in Table 3. Younger participants reported higher total activity, light activities, sports, and occupational activities and lower gardening scores than the older respondents. Amputees reported higher total activity, higher sports and occupational activity, and lower gardening scores. Respondents with normal BMI seemed to be more active than other respondents. Overweight respondents had higher scores in light activities only. Hemiplegic respondents reported higher gardening scores. Auditorily impaired respondents reported higher light activity scores. 

**Table 3:** Total and subcategory scores for the PASIPD by descriptive characteristics.

Variable	Total score	Factor 1	Factor 2	Factor 3	Factor 4
Age group (years)					
<50 (112)	18.4 ± 13.9**	2.2 ± 3.8	13.5 ± 11.5***	0.9 ± 2.6*	1.9 ± 1.5
≥50 (86)	13.9 ± 17.7	2.5 ± 5.3	7.7 ± 12.7	1.8 ± 3.6	1.6 ± 1.1
Disability†					
Amputee (n: 106)	17.8 ± 18.0	3.1 ± 5.2***	11.6 ± 13.4	1.3 ± 3.1	1.7 ± 1.4**
Hemiplegia (n: 24)	15.6 ± 12.7	3.4 ± 5.4	9.5 ± 10.3	2.3 ± 3.9	1.3 ± 1.0
CP (n: 4)	2.2 ± 1.9	0.3 ± 0.6	1.0 ± 1.8	0.0 ± 0.0	0.8 ± 1.2
Auditory impairment (n: 64)	15.3 ± 12.6	0.8 ± 1.6	11.1 ± 11.3	1.1 ± 2.8	2.2 ± 1.3
Weight					
Underweight (BMI <18.5) (n: 8)	10.9 ± 15.3	0.8 ± 1.81	6.7 ± 9.4	1.6 ± 4.6	1.7 ± 1.2
Normal (BMI 18.5–24.9) (n: 130)	17.8 ± 16.3	2.5 ± 4.4	12.3 ± 12.8	1.4 ± 3.2	1.7 ± 1.3
Overweight (BMI 25–29.9) (n: 60)	14.2 ± 14.3	2.3 ± 5.0	8.7 ± 11.3	1.0 ± 2.7	2.0 ± 1.5
Total (n: 198)	16.4 ± 15.8	2.3 ± 4.5	11.0 ± 12.3	1.3 ± 3.1	1.8 ± 1.4

*P < 0.05, **P < 0.01, ***P < 0.001 †CP group was excluded for statistical comparison due to the limited number of patients.

## 4. Discussion

The Turkish version of the PASIPD used in the current study can be considered to be statistically reliable and valid for measuring the physical activity level of disabled people. Unlike the original study of the questionnaire, in which 5 latent factors were determined, the statistical analysis of the current study determined 4 latent factors. When compared to the factors of the original questionnaire, the sport and recreation factors were divided as 2 separated factors, while in our study, the sport factor held both sports and occupation. Although all the questions did not need to be adapted, the socioeconomic and sociocultural characteristics of the community where the disabled individuals were living did not allow for some participants to answer some questions (question nos. 5, 6, 9, 10, 11). For this reason, two of the factors were combined into one. Since the scoring of the questionnaire has a unique calculation for each question, there will not be any defect in total scoring. For the aim of our study, during clinical decisions the specific score of each question should also be considered.

Older participants had statistically signiﬁcant lower total PASIPD scores than younger participants, but the reverse was found in the scores for heavy household activities, care for another person, and gardening. The age-related decline in lean body mass is well known and is primarily due to atrophy of muscle cells. Since aging is associated with a progressive decline in bone and skeletal muscle mass, the force-generating capacity leads to a gradual decrease in the physical activity level [23,24]. 

Amputees were seen to be the most active disability group in the study. The amputees participating in this study exercised regularly, and the least active group was the auditorily impaired participants. This is a potential question for the future: to find the reason for the difference in physical activity levels between those disabled groups. 

Similarly, in the current study, disabled respondents with normal BMI were more active. A surprising result was that hemiplegic participants had the highest scores for gardening activity, which could indicate that hemiplegic individuals spend more time outdoors. Overweight and auditorily impaired participants were the most inactive groups in the current study. The test and retest results showed no statistical significance. With the ICC scores (r: 1, Spearman’s rank correlation coefficient), it was statistically proven that the Turkish version of the PASIPD can be effectively used in clinical settings. 

The test and retest results showed perfect reliability. The ICC and Spearman rank correlation coefficients were 1.0 (P < 0.001) since all the respondents reported the same answers in the test and retest questionnaires. This may be due to the short time interval between the two interviews (1–3 days apart).

A limitation of this study is that the results were obtained from participants who were physically active but not regularly taking part in any sports. Questions about other demographic characteristics that may affect the physical activity level were not known and should therefore be included in further studies. 

In conclusion, the results of this study showed that the Turkish version of the PASIPD is a valid and reliable tool for the quantification of the physical activity level of disabled individuals in a Turkish population. Physiotherapists and clinicians can use this questionnaire to measure the physical activity level of disabled individuals in a simple and inexpensive manner.
